# Editorial: Microorganisms in polar regions: understanding their survival strategies for a sustainable future

**DOI:** 10.3389/fmicb.2024.1442926

**Published:** 2024-06-24

**Authors:** Trista J. Vick-Majors, Shiv Mohan Singh, Prashant Kumar Singh

**Affiliations:** ^1^Department of Biological Sciences, Michigan Technological University, Houghton, MI, United States; ^2^Department of Botany, Banaras Hindu University, Varanasi, India; ^3^Department of Biotechnology/Life Sciences, Pachhunga University College (A Constituent College of Mizoram University), Aizawl, Mizoram, India

**Keywords:** psychrophile, Arctic, Antarctic, adaptation, biotechnology

The Research Topic, *Microorganisms in Polar Regions: Understanding Their Survival Strategies for a Sustainable Future*, compiles contributions exploring aspects of polar microbiology ranging from specific adaptations to low-temperature environments to aspects of microbial community ecology in polar glaciers and aquatic environments. The Arctic and Antarctic are being transformed due to climate change, and ecosystem responses to environmental change are mediated by the metabolic activities of the microorganisms that inhabit them.

In the Research Topic, 11 articles focused on the unique adaptations and lifestyles of microbial life in the polar regions inform our understanding of their potential responses to changing environmental conditions, and some of the ways their adaptations can be harnessed for biotechnological development to support a sustainable future.

Dutta et al. investigated what drives the distribution of microbial ecological function along the coastal western Antarctica Peninsula. The authors subjected DNA samples collected as part of the Palmer Long Term Ecological Research project to metagenomic sequencing. They developed a pipeline for metagenomic data processing (iMAGine) that allowed them to uncover differences in microbial community functional potential across depth and regional profiles. Their data suggest metabolically flexible microorganisms are key to sustaining microbial community function under changing environmental conditions.

Vannier et al. examined metagenomes from Skaftárkatlar, two geothermally and volcanically influenced subglacial lakes beneath the Vatanjökull ice cap in Iceland. These environments are unique in blending geothermal and volcanic inputs with subglacial isolation (the ice cap is ~250 m thick). The authors found that chemolithoautotrophic metabolisms dominate the subglacial ecosystem and that enrichment cultures did not include the dominant organisms and metabolisms. This underscores the difficulty of working with these unique samples and the importance of conducting *in situ* investigations to understand microbial life in these environments.

Shaffer et al. demonstrated the utility of examining individual genomes by focusing on characterizing a red-pigmented, motile strain of the bacterium *Massilia frigida*. The organism was isolated from a microbial mat in the Don Juan Pond basin in Wright Valley, Antarctica. Don Juan Pond is a hypersaline environment, and they were able to identify not only metabolic pathways and genes related to salt and cold tolerance, but also to test the effect of UV-A radiation on the organism. The isolate produced prodigiosin, a bactericidal, nematocidal, and UV-protective compound, which may have implications for organismal interactions and resilience to UV exposure in microbial mats.

Prekrasna-Kviatkovska et al. focused on indirect effects of climate change in the rapidly changing central maritime region of the Antarctic. Moss banks in the region are capable of accumulating peat, but this process has been impacted by the southward expansion of Gentoo penguin colonies. The authors examined the effect of Gentoo penguin colonization pressure on the chemical composition of and microbial communities associated with moss banks. The results showed that penguin colonization was associated with phylum-level shifts in peat microbiota, and increases in peat pH and soluble nitrogen and phosphorus concentrations. Overall, the expansion of penguin colonies was associated with loss of acidiphilic microbiomes normally associated with moss banks.

Singh et al. examined cryoconite associated with the Hamtah Glacier, one of more than 50,000 glaciers located in India's Himalayas. Through the use of cultivation-dependent, cultivation-independent, and chemical methods, they assessed the diversity and functional potential of bacterial life and elemental composition in Hamtah cryoconite. The study revealed a diverse microbial community including organisms containing cold-active enzymes, which may be biotechnologically relevant.

In the review by Dopson et al., the focus was on eurypsychrophilic acidophiles which inhabit cold, acidic sites ranging from acid rock drainage that can be found in the South Shetland Islands, Antarctica, to sulfidic sediments of the Baltic Sea coastal region, and occur on extra-planetary bodies such as the Jovian moons. The work provided evolutionary, environmental, biotechnological, and exobiological perspectives on five characterized low-temperature adapted acidophiles whose capacities to impact the composition of metallic compounds make them biogeochemically and biotechnologically relevant.

Shrestha et al. examined the genome of the extremophilic bacterium, *Glaciimonas* sp. PAMC28666, which was originally isolated from Antarctic soil. In a laboratory-based experiment, they examined the survival of PAMC28666 in response to freezing and thawing. They revealed enhanced expression of *trehalose 6-phosphate synthase (otsA)* and *trehalose 6-phosphate phosphatase (otsB)* on shifting temperature from thawing to freezing. They also determined that the overexpression of *otsAB* led to enhanced production of trehalose, which may be important in cold adaptation.

Juchem et al. demonstrated the effect of prolonged exposure to dark conditions mimicking the seasonally fluctuating light conditions in Antarctica in benthic diatoms important in biogeochemical cycling and primary productivity. They isolated four distinct species of benthic diatoms, of which *Planothidium wetzelii* sp. nov. was newly described. After being subjected to a dark incubation period of 3 months, adaptive strategies were identified in the diatoms. They demonstrated chloroplast degradation and a decline in storage lipid content; however, no species exhibited a change in photosynthetic performance. They concluded that survival strategies manifested as biochemical cell biological changes allow benthic diatoms to survive the Antarctic night.

Ramasamy et al. reviewed the adaptive mechanisms of Antarctic marine bacteria to cope with climatic change at the mosphological, physiological, and molecular levels. They also addressed recent advances in “omics” techniques and machine learning approaches to generate molecular understanding of psychrophiles and bacterial diversity. Finally, the manuscript also described cold-adapted enzymes and compounds which may have commercial potential in biotechnology.

Liu et al. described current knowledge and recent advances related to enzymes derived from cold-adapted microorganisms. Their uses and the catalytic processes they are involved in were discussed, as were methods of molecular modification that may increase the utility of a given enzyme. Overall, the review set the stage for future research into the use of cold-adapted enzymes.

Xiao et al. isolated and identified a symbiotic bacterium, *Serratia myotis* L7-1, from an Antarctic fish, *Trematomus bernacchii*. On whole genome sequencing, they found that its genome encoded for carbohydrate-active enzymes (CAZymes), biosynthetic pathway genes, stress-responsive genes, antibiotic-resistant genes (ARGs), and a complete type IV secretion system. Based on sequencing results, they performed bioactivity-guided fractionation and identified *Serratia myotis* L7-1 as having antibacterial and antitumor activities.

This Research Topic of papers demonstrates the importance of polar microbes in both biotechnological and ecological contexts, and highlights work still to be done in understanding the diversity of microbial life in the polar regions. The research being conducted to uncover the biodiversity and understand the survival strategies of these microbes also sets the stage for biotechnological applications in the future, which may benefit from the unique adaptations of polar microbes. Together, the work in this Research Topic underscores the benefits of combining molecular information, machine learning techniques, and ecological data to futher our understanding of the microbiology of these unique and rapidly changing environments.

## Author contributions

TV-M: Writing – original draft, Writing – review & editing. SS: Writing – original draft, Writing – review & editing. PS: Writing – original draft, Writing – review & editing, Project administration.

